# Apolipoprotein B/apolipoprotein A1 ratio and mortality among incident peritoneal dialysis patients

**DOI:** 10.1186/s12944-018-0771-z

**Published:** 2018-05-17

**Authors:** Xiaojiang Zhan, Yanbing Chen, Caixia Yan, Siyi Liu, Lijuan Deng, Yuting Yang, Panlin Qiu, Dan Pan, Bingxiang Zeng, Qinkai Chen

**Affiliations:** Department of Nephrology, The First Affiliated Hospital of Nanchang University, 17# yongwai street, Nanchang, 330006 Jiangxi China

**Keywords:** Apolipoprotein, Cardiovascular event, Cohort study, Mortality, Peritoneal dialysis

## Abstract

**Background:**

To investigate the association between the ratio of apolipoprotein B (apo B) / apolipoprotein A1 (apo A1) with all-cause mortality and cardiovascular events in peritoneal dialysis (PD) patients.

**Methods:**

Eight hundred and sixty incident PD patients were enrolled from November 1, 2005, to February 28, 2017, and followed until May 31, 2017. Outcomes were all-cause mortality and cardiovascular events. Associations between the apo B/apo A1 ratio with all-cause mortality and cardiovascular events were evaluated using multivariable-adjusted Cox models.

**Results:**

Of the 860 patients, the mean age was 49.9 ± 14.5 years, 57.6% were men, and 19.3% were diabetic patients. The median apo B/apo A1 ratio was 0.65 (range: 0.22–2.24). During a median follow-up period of 27 months (interquartile range, 13 – 41 months), 202 deaths, and 145 cardiovascular events were recorded. After adjustment for age, sex, body mass index, diabetes, cardiovascular disease, systolic blood pressure, total Kt/V, estimated glomerular filtration rate, hemoglobin level, neutrophil to lymphocyte ratio and albumin, triglyceride, and cholesterol, as well as the use of lipid-lowering agents, the highest apo B/apo A1 ratio tertile was significantly associated with a hazard ratio for all-cause mortality of 1.60 (95% CI: 1.02 to 2.49, *P* = 0.040) and for cardiovascular events of 2.04 (95% CI: 1.21 to 3.44, *P* = 0.008).

**Conclusion:**

An increased apo B/apo A1 ratio was independently associated with all-cause mortality and cardiovascular events in PD patients.

## Background

Peritoneal dialysis (PD) patients have an increased risk of morbidity and mortality associated with cardiovascular disease (CVD), which is, at least in part, due to lipid abnormalities, typically called uremic dyslipidemia [1]. Patients typically present with a decrease of the high-density lipoprotein cholesterol (HDL-C), and an increase of triglyceride (TG) and lipoprotein (a) [Lp (a)], with relatively normal or even lower levels of low-density lipoprotein cholesterol (LDL-C) [2].

LDL-C is widely recognized as the major atherogenic lipoprotein and is considered the primary therapeutic target for coronary heart disease [3]. Furthermore, multiple lines of evidence indicate that other TG-rich lipoproteins, including intermediate density lipoprotein (IDL) or very low-density lipoprotein (VLDL) carry atherogenic potential as well [4]. It has been reported that approximately 50% of patients with abnormalities of atherogenic lipoprotein had an increased risk for the development of CVD [5]. This led to the consideration of non-HDL cholesterol as another important target for hyperlipidemia treatment. It is well known that, lipid-transporting apolipoprotein is an essential structural component of the lipoprotein cholesterol [6], VLDL, IDL and LDL cholesterol can each carry only a single apolipoprotein B (apo B) [7] and this characteristic can be used to reliably reflected the number of atherogenic lipoproteins in the plasma. In contrast, apo A1 has a strong association with HDL levels and accounts for approximately 60-70% of the total apolipoproteins in HDL-C, the plasma content of apo A1 represents the total of antiatherogenic particles [8]. Therefore, the ratio of apo B to apo A1 (apo B/apo A1) reflects the balance of proatherogenic and antiatherogenic particles.

Recently, Sato et al. [9] indicated that a higher ratio of the apo B/apo A1 was associated with higher risk of all-cause and CVD-related mortality in patients on hemodialysis (HD). However, the lipid profile of PD patients was different from HD patients. In particular the lipid profile is more atherogenic in nature with more altered dyslipidemia when compared with those in HD patients [10]. Moreover, the association between the apo B/apo A1 ratio and mortality in PD patients remains uncertain. Therefore, we hypothesized that a higher ratio of apo B/apo A1 is a critical parameter to predict the risk of cardiovascular events and all-cause mortality in PD patients. In this longitudinal cohort study, we assessed the associations between the apo B/apo A1 ratio with all-cause mortality and cardiovascular events in PD patients, and followed the patients for a median of 27 months at our PD center.

## Methods

### Study population and data collection

We studied all incident patients who used PD as their first renal replacement treatment modality and were followed up at the PD center of The First Affiliated Hospital, Nanchang University, Jiangxi, China from November 1, 2005, to February 28, 2017. Inclusion criteria were age ≥ 18 years at the start of PD and survival for at least 90 days from the first PD therapy. The patients who were catheterized in other hospitals, transferred from permanent HD, or failed renal transplantation were excluded in this study. The study was conducted in compliance with the ethical principles of the Helsinki Declaration (https://jamanetwork.com/journals/jama/fullarticle/1760318) and approved by the Human Ethics Committees of Nanchang University.

All patients were followed up until cessation of PD, death, or May 31, 2017. Baseline demographic data included age, sex, primary cause of end-stage renal disease (ESRD), and presence of diabetes and CVD. Clinical and biochemical data at the initiation of PD included body mass index, blood pressure, medication use, hemoglobin, serum albumin, serum creatinine, blood urea nitrogen, total cholesterol (CHOL), TG, HDL-C, LDL-C, Apo B, Apo A1, Lp (a). Immunoturbidimetric method was used to measure apo B and apo A1 concentrations. All baseline data were obtained during the first 1–3 months of PD. Baseline residual renal function was assessed by eGFR using the Chronic Kidney Disease Epidemiology Collaboration creatinine equation. Cardiovascular events were defined by the first occurrence of myocardial infarction, stroke, heart failure, hospitalization for unstable angina, peripheral vascular event, sudden death, death associated with a cardiovascular procedure, or death due to aneurysm dissection or rupture, fatal pulmonary embolism, or death due to other or unknown cardiovascular cause [11] after the onset of PD, and was determined by the PD follow-up panel composed of PD primary nurses and professors.

### Statistical analyses

Patients with apo B/apo A1 ratio were classified into tertiles (Ts): T1 ≤ 0.545, T2 = 0.545-0.769, T3>0.769. Participant characteristics were calculated by Ts of apo B/apo A1. Results were expressed as frequencies and percentages for categorical variables, means and standard deviations (SDs) for normally distributed continuous variables, and medians and interquartile ranges for continuous variables not normally distributed. Chi-squared, one-way ANOVA, or Kruskal–Wallis tests were used to test for differences in categorical or continuous factors among different categories of apo B/apo A1. Survival times were estimated from Kaplan–Meier curves, and differences in survival probabilities among groups were assessed using the log-rank test. The associations between apo B/apo A1 ratio and all-cause mortality and cardiovascular events were examined in Cox proportional hazards models. The censored data included switching to HD, renal transplantation, moving to another center, declining additional treatment, loss to follow-up, or still at our PD center on May 31, 2017. Unadjusted associations were first examined followed by adjustments for age and sex, diabetes and CVD, systolic blood pressure (SBP), body mass index (BMI), total urea clearances (tKt/V) and eGFR, hemoglobin, neutrophil to lymphocyte ratio (N/L), albumin. Next, TG, CHOL as well as lipid lowering agents were added to examine whether apo B/apo A1 ratio was independently associated with mortality and cardiovascular events. Covariates with *P* < 0.05 in the univariate Cox analyses or thought to be clinical significant were chosen for multivariate Cox proportional hazards regression. The results were expressed as the hazard ratio (HR) and 95% confidence interval (95% CI). All descriptive and multivariate analyses were conducted using SPSS version 22.0 (SPSS, Inc., Chicago, IL). A value of *P* < 0.05 was considered statistically significant.

## Results

### Baseline patient characteristics

**Fig. 1:**
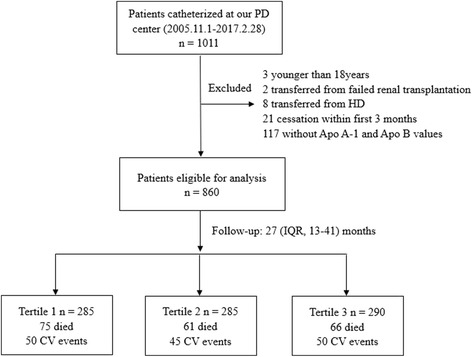
Enrollment flow chart for analysis. PD, peritoneal dialysis; HD, hemodialysis; Apo A1, apolipoprotein A1;Apo B, apolipoprotein B; IQR, interquartile range; CV, cardiovascular

A total of 1011 incident PD patients were recruited and monitored in our hospital. Thirty-four of the patients were excluded by our experimental criteria and listed as below: 3 subjects were under 18 years of age, 2 subjects were transferred from failed renal transplantation, 8 subjects were transferred from permanent HD, and 21 subjects were treated with PD for less than 3 months. The remaining 977 patients were enrolled. Of the 977 patients, 117 patients did not measure baseline levels of apolipoproteins. Finally, 860 patients were found to be eligible for analysis. During the PD procedure, the conventional PD dialysis fluids include 1.5% or 2.5% dextrose and the twin-bag system was applied for all PD patients. For the management of dyslipidemia in the patients, lipid-lowering agents were formulated and given to the patients (10.3%). As shown in Fig. 1, the mean (± SD) age of the subjects was 49.9 ± 14.5 years (57.6% were men). 10.1% of patients had prior CVD and 19.3% of patients had diabetic history. The leading cause of ESRD was chronic glomerulonephritis, which accounted for 64.3% of the group, followed by diabetic nephropathy (16.3%) and hypertension (12.8%).

### Apo B/apo A1 tertiles

The baseline of the apo B/apo A1 ratio ranged between 0.22 and 2.24 (interquartile range = 0.50–0.85 U/L, mean = 0.65) and the characteristics of the patients stratified by tertiles of the apo B/apo A1 ratio are displayed in Table 1. As shown in Table 1, patients with a higher apo B/apo A1 ratio presented with diabetic tendency, and increased BMI, tKt/V, eGFR, N/L, hemoglobin, TG, CHOL, LDL, apo B and Lp (a), and decreased levels of HDL and apo A1 (*P* < 0.05). No significant differences among age, sex, CVD, blood pressure, albumin, and lipid-lowering agents use were observed (Table 1).

**Table 1:** Baseline characteristics of individuals stratified by tertiles of apo B/apo A1 ratio

Variables	Apo B/Apo A1 Ratio Tertiles			Total	*P* Value
	≤0.545 (*n* = 285)	0.545 − 0.769 (n = 285)	>0.769 (*n* = 290)		
Age (yr)	50.9 ± 14.8	48.7 ± 14.3	50.0 ± 14.5	49.9 ± 14.5	0.200
Men (%)	164 (57.5)	172 (60.4)	159 (54.8)	495 (57.6)	0.408
Body mass index (kg/m^2^)	21.0 ± 3.2	22.2 ± 3.5	22.6 ± 3.4	21.9 ± 3.4	< 0.001
Diabetes (%)	46 (16.1)	50 (17.5)	70 (24.1)	166 (19.3)	0.034
CVD (%)	25 (8.8)	25 (8.8)	37 (12.8)	87 (10.1)	0.186
Systolic pressure (mmHg)	145 ± 27	148 ± 26	147 ± 26	147 ± 26	0.541
Diastolic pressure (mmHg)	87 ± 17	90 ± 15	88 ± 15	87 ± 17	0.067
Total Kt/V	2.01 (1.43, 2.53)	2.26 (1.79, 2.69)	2.23 (1.81, 2.80)	2.18 (1.68, 2.70)	< 0.001
eGFR (ml/min per 1.73 m^2^)	2.66 (1.54, 4.09)	3.59 (2.02, 5.82)	3.85 (1.89, 6.07)	3.26 (1.78, 5.56)	< 0.001
Hemoglobin (g/L)	76.65 ± 16.48	79.71 ± 16.19	80.74 ± 16.75	79.04 ± 16.55	0.009
N/L	3.27 (2.37, 4.75)	3.50 (2.53, 5.53)	3.86 (2.61, 5.20)	3.58 (2.50, 5.12)	0.028
Albumin (g/L)	35.90 ± 5.00	35.70 ± 5.31	34.50 ± 5.29	35.36 ± 5.23	0.848
Total cholesterol (mmol/L)	3.56 (3.05, 4.30)	4.03 (3.46, 4.80)	4.55 (3.84, 5.40)	4.06 (3.34, 4.89)	< 0.001
Triglyceride (mmol/L)	1.00 (0.73, 1.36)	1.27 (0.91, 1.72)	1.68 (1.14, 2.21)	1.28 (0.89, 1.78)	< 0.001
Low density lipoprotein (mmol/L)	1.90 (1.40, 2.30)	2.31 (1.89, 2.93)	2.81 (2.24, 3.46)	2.30 (1.83, 2.94)	< 0.001
High density lipoprotein (mmol/L)	1.20 (0.96, 1.53)	1.14 (0.92, 1.42)	0.98 (0.81, 1.16)	1.08 (0.89, 1.37)	< 0.001
Apo B (g/L)	0.60 (0.50, 0.70)	0.80 (0.70, 0.90)	1.07 (0.90, 1.41)	0.80 (0.63, 1.00)	< 0.001
Apo A1 (g/L)	1.38 (1.22, 1.59)	1.23 (1.10, 1.40)	1.09 (0.98, 1.23)	1.22 (1.07, 1.41)	< 0.001
Lipoprotein (a) (mg/L)	284 (141, 514)	303 (160, 501)	409 (226, 663)	328 (172, 586)	< 0.001
Lipid-lowering agents use (%)	29 (10.2)	36(12.6)	24(8.3)	89(10.3)	0.228

*CVD* cardiovascular disease, *N/L* neutrophil to lymphocyte ratio, *Apo B*, apolipoprotein B, *Apo A1* apolipoprotein A1

*P* < 0.05 is considered statistically significant

### The correlation of lipid parameters

As shown in Table 2, the parameter apo A1 was strongly correlated with HDL and the apo B/apo A1 ratio (*r* = 0.60 and − 0.51, respectively). Apo B was strongly correlated with LDL and the ratio of apo B/apo A1 (*r* = 0.74, and 0.81, respectively). On the other hand, the ratio of N/L was negatively correlated with apo A1 (*r* = − 0.10) but not with apo B (*r* = 0.04; Table 2).

**Table 2:** Correlation between Apo B/Apo A1 and parameters of lipid and inflammation

	Apo B/Apo A1	CHOL	TG	LDL	HDL	Apo A1	Apo B	Lp (a)
CHOL	0.40^a^							
TG	0.45^a^	0.38^a^						
LDL	0.51^a^	0.88^a^	0.32^a^					
HDL	−0.29^a^	0.43^a^	−0.30^a^	0.28^a^				
Apo A1	−0.51^a^	0.35^a^	−0.09^b^	0.20^a^	0.60^a^			
Apo B	0.81^a^	0.72^a^	0.46^a^	0.74^a^	0.05^c^	0.03^c^		
Lp (a)	0.16^a^	0.27^a^	0.03^c^	0.28^a^	0.08^b^	0.09^a^	0.25^a^	
N/L	0.08^b^	−0.00^c^	−0.01^c^	−0.01^c^	-0.02^c^	-0.10^a^	0.04^c^	0.03^c^

*CHOL* cholesterol, *TG* triglyceride, *LDL* low density lipoprotein, *HDL* high density lipoprotein, *Apo A1* apolipoprotein A1, *Apo B* apolipoprotein B, *Lp (a)* lipoprotein (a), *N/L* neutrophil to lymphocyte ratio

^a^Correlation is significant at the 0.01 level (two-tailed)

^b^Correlation is significant at the 0.05 level (two-tailed)

^c^Correlation is not significant

### The correlation of Apo B/apo A1, all-cause mortality, and cardiovascular events

**Fig. 2:**
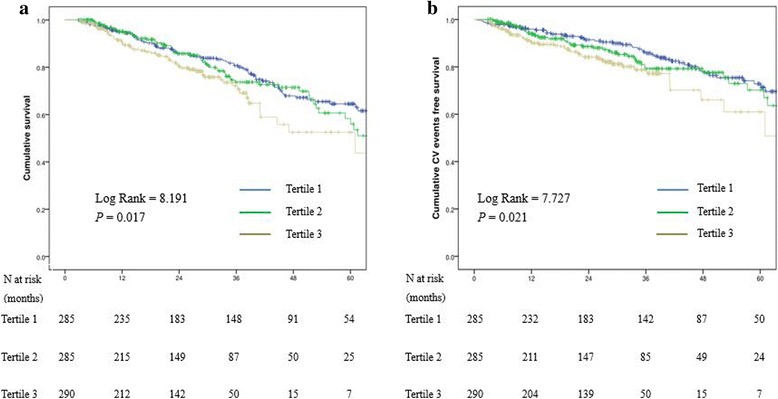
Survival curves for patients stratified by Apo B/Apo A1 ratio. **a** All-cause mortality-free survival curves. **b** Cardiovascular events-free survival curves

In this study, the median follow-up period was 27 months (interquartile range = 13–41 months). By the end of the study, 202 patients (23.5%) were died and recorded. Fifty-two patients (6.0%) had received kidney transplantation, 127 patients (14.8%) were transferred to HD, 6 patients (0.7%) transferred to other PD centers, 20 patients (2.3%) patients discontinued follow-up, and the remaining 453 patients (52.7%) were still followed in our PD center. Of the 202 deaths, 108 were caused by CVD (53.5%), 16 were caused by infectious disease (7.9%), 3 were due to malignancy (1.5%), 16 were caused by cachexia (7.9%), and 19 and 40 cases (9.4 and 19.8%, respectively) were caused by other and unknown reasons, respectively. The correlation between all-cause mortality and the apo B/apo A1 ratio was evaluated using Kaplan–Meier methods, which are shown in Fig. 2. The survival rate was also estimated at the end of 1, 3, and 5 years as follows: 94.9, 80.7, and 64.5%, in the T1 group; 95.1, 73.7, and 58.3%, in the T2 group; and 90.6, 72.2, and 52.5% in the T3 group. The results showed that the total survival rate in the T3 group was significantly lower compared to the T1 and T2 groups (*P* = 0.017) (Fig. 2a). The cardiovascular events-free survival rate at the end of 1, 3, and 5 years was (respectively) 96.0, 85.8, and 72.8% in the T1 group; 94.3, 79.2, and 70.2% in the T2 group; and 90.4, 78.8, and 61.0% in the T3 group. Similarly, the cardiovascular events-free survival rate in the T3 group was lowest among these groups (*P* = 0.021) (Fig. 2b). The association between the apo B/apo A1 ratio and all-cause mortality and cardiovascular events was determined using Cox regression analysis. As shown in Table 3, despite the adjustment in models 2 and 3, the apo B/apo A1 ratio was still associated with all-cause mortality and cardiovascular events. In model 3, the HRs and 95% CIs for tertile 3 versus tertile 1 were: HR, 1.60 (95% CI: 1.02 to 2.49), and HR, 2.04 (95% CI: 1.21 to 3.44), for all-cause mortality and cardiovascular events, respectively. These results suggest that apo A1 was correlated with all-cause mortality in models 2 and 3, and cardiovascular events in models 1 and 2. Apo B was not correlated with either parameter in models 1-3 (Table 3).

**Table 3:** The associations of apo A1, apo B, and the apo B/apo A1 ratio (Tertile3 Vs Tertile1) with all-cause mortality and cardiovascular events

Variables	Apo A1		Apo B		Apo B/Apo A1 ratio^d^	
	HR (95% CI)	*P* Value	HR (95% CI)	*P* Value	HR (95% CI)	*P* Value
All-cause mortality						
Model 1^a^	0.64 (0.39 - 1.08)	0.094	1.22 (0.78 - 1.90)	0.384	1.62 (1.15 - 2.29)	0.005
Model 2^b^	0.47 (0.25 - 0.89)	0.020	0.92 (0.52 - 1.65)	0.789	1.59 (1.05 - 2.43)	0.031
Model 3^c^	0.48 (0.24 - 0.94)	0.033	0.73 (0.31 - 1.72)	0.466	1.60 (1.02 - 2.49)	0.040
Cardiovascular events						
Model 1^a^	0.47 (0.25 - 0.90)	0.022	1.04 (0.60 - 1.80)	0.900	1.76 (1.17 - 2.63)	0.006
Model 2^b^	0.39 (0.18 - 0.83)	0.015	0.80 (0.40 - 1.61)	0.535	1.72 (1.05 - 2.81)	0.030
Model 3^c^	0.44 (0.19 - 1.02)	0.055	1.14 (0.42 - 3.10)	0.795	2.04 (1.21 - 3.44)	0.008

*Apo A-1* apolipoprotein A-1, *Apo B* apolipoprotein B, *HR* Hazard ratios, *95% CI* 95% confidence intervals

^a^Model 1: unadjusted

^b^Model 2: adjusted for age, sex, BMI, diabetes, CVD, SBP, eGFR, tKt/V, hemoglobin, N/L, and albumin

^c^Model 3: model 2 adjusted for cholesterol, triglyceride, and lipid-lowering agents use

^d^Tertile 3 versus tertile 1

## Discussion

In this retrospective cohort study, we identified the correlation between the serum apo B/apo A1 ratio and PD patient characteristics. We found, for the first time, that a higher apo B/apo A1 ratio is strongly correlated with increased all-cause mortality and cardiovascular events in PD patients. Consistent with the empirical results from HD patients and non-CKD patients [9, 12–15], the apo B/apo A1 ratio can be used as an important risk indicator for clinicians in various fields, including dialysis.

Apo B is a key basal unit of atherogenic lipoprotein including VLDL, IDL, and LDL. Since each of these lipoproteins detects only one apo B molecule, apo B measurements have been used to determine the precise number of the atherogenic lipoproteins in patients [7]. Previous studies indicated that apo B was a strong predictor of mortality and cardiovascular risk [9, 16]. Contrary to these studies, we did not find a significant correlation between apo B and these events. Some differences between these studies and our results are worth noting. First, in our cohort study, the follow-up time was approximately 2 years, whereas other studies have follow-up times of more than 7 years. Second, we enrolled incident PD patients in our study, whereas Sato et al. [9] assessed the changes apo B in prevalent HD patients. These possibilities may cause the variations between our results and others, and these contradictions should be further elucidated in future studies.

In contrast to apo B, apo A1 is a main constituent of HDL. Although each HDL particle contains five apo A1 molecules, systemic apo A1 levels have been used as an indicator for HDL cholesterol concentrations [17]. The relationship between apo A1 and cardiovascular events remains controversial. A large meta-analysis of prospective studies in the general populations shows an inverse association between apo A1 and incident coronary heart disease [18]. However, Zhu et al. [16] found that apo A1 was not correlated with cardiovascular events in the general population. In the diabetes dialysis study in German, apo A1 failed to predict cardiovascular events or all-cause mortality [19]. Sato et al. [9] also found that apo A1 was not independently associated with CVD-related and all-cause mortality. Interesting, in this study, we found that apo A1 had a higher association with the prevention of mortality in adjusted models, and cardiovascular events in model 1 and model 2, with a marginal significance for cardiovascular events in model 3. We speculate that the difference in population and follow-up time may be important factors for apo A1 adjustment and application.

The apo B/apo A1 ratio is a typical marker for the atherogenic /antiatherogenic condition, and a higher apo B/apo A1 ratio represents progression in the atherosclerotic state. Previous studies have suggested that a high apo B/apo A1 ratio strongly correlates with pathophysiological markers of atherosclerosis defined by coronary angiography [8, 20], and calcium scores of the coronary arteries [21], and by ultrasound techniques such as the thickness of carotid arteries [16, 22, 23], endothelial dysfunction [24] and existence of femoral plaques [25]. In addition, a high apo B/apo A1 ratio can also be used to predict risk of carotid plaque development [26] and major adverse cardiovascular events [14, 27–29]. Our findings are in agreement with these studies and show that the apo B/apo A1 ratio is independently associated with cardiovascular events.

Although the apo B/apo A1 ratio is considered as a risk predictor for CVD mortality in the general population [14, 30], to date, few studies have used the apo B/apo A1 ratio to predict the mortality of dialysis patients. The results were also conflicting. For example, Sato et al. [9] showed that a higher apo B/apo A1 ratio was associated with an increased risk of all-cause and CVD-related mortality in prevalent HD patients. However, in that study, BMI, which has been associated lower mortality risk, was not adjusted. Another study demonstrated that the baseline apo B/apo A1 ratio did not correlate with 4-year mortality [31]. In that study, after adjustment for confounding factors, a higher apo B/apo A1 ratio predicted first-year survival and the next 3 years mortality, and an increased ratio during the first year was also associated with a survival advantage in dialysis patients. However, this association lost significance after adjustment for the protein-energy wasting index. In this study, our findings extended this observation and indicated that the increased risk of all-cause mortality is associated with an increase of the apo B/apo A1 ratio, independent of BMI and N/L, in PD patients. It was reported that the lipid profile was different in PD and HD, and the apo B/apo A1 ratio was higher in patients on HD than patients receiving PD [32], which is supported by the previous study by Sato et al. [9] and our results. However, our study indicated that the association between the apo B/apo A1 ratio and mortality in PD patients was similar to the previous study in HD patients [9].

The present study also has limitations to discuss. First, this is a retrospective study, which can only lead to associations but not causality. Second, the number of patients is relatively small. Third, in this study, we only applied the baseline data but did not exclude patients who had already taken lipid-lowering medications, nor considered the data changes during the follow-up period. Fourth, because of the limited sample size, the potential risk factors were not all adjusted in this cohort study. Hence, the effects of residual confounding factors cannot be eliminated completely. Our future studies will address the improvement of these issues.

## Conclusions

In summary, despite conflicting evidence of lipid effects on the prognosis of PD patients, our results found significant associations between the apo B/apo A1 ratio, cardiovascular events, and all-cause mortality in PD patients. The apo B/apo A1 ratio can serve as a risk marker for the risk of the CVDs and mortality in PD patients.
